# Diagnostic performance of ultrasound with computer-aided diagnostic system in detecting breast cancer

**DOI:** 10.1016/j.heliyon.2023.e20712

**Published:** 2023-10-06

**Authors:** Pengjie Song, Li Zhang, Longmei Bai, Qing Wang, Yanlei Wang

**Affiliations:** a Department of Ultrasound, Gangkou Hospital, Qinhuangdao, Hebei, 066000, China; b Department of Ultrasound, Peking University Third Hospital Qinhuangdao Hospital, Qinhuangdao, Hebei, 066000, China

**Keywords:** Breast mass, Breast cancer, Ultrasound, Computer-aided diagnosis

## Abstract

**Purpose:**

This study aims to examine the performance of breast ultrasound with a computer-aided diagnostic (CAD) system in detecting malignant breast cancer compared to conventional ultrasound and investigate the effects on smaller tumor sizes (≤20 mm).

**Methods:**

This retrospective analysis included 123 patients with breast masses between March 2021 and July 2023. By using pathology results from biopsies or surgeries as the gold standard, we calculated and compared the diagnostic performances of conventional ultrasound and CAD, including sensitivity, specificity, positive predictive value, negative predictive value, accuracy, and area under the receiver operating characteristic curve (AUC). A subgroup analysis of masses ≤20 mm in size was performed.

**Results:**

Twenty-seven patients were pathologically diagnosed with malignant breast cancer. CAD had a higher specificity (92.71 % vs. 62.5 %) and accuracy (93.5 % vs. 69.92 %) than conventional ultrasound. The AUC of CAD was significantly greater than that of conventional ultrasonography (0.9450 vs. 0.7940, p < 0.0001). The agreement between the CAD and pathology results was almost perfect (kappa = 0.82, p < 0.0001). In patients with masses ≤20 mm, the effect was consistent: CAD had higher specificity (91.43 % vs. 51.43 %), higher accuracy (90.70 % vs. 58.14 %), and a higher AUC (0.8946 vs. 0.6946, p < 0.0001) than conventional ultrasound. Thirty-one downgrades were observed in BI-RADS 4A and 4B based on CAD, all of which were proven to be benign.

**Conclusion:**

Compared to conventional breast ultrasound, CAD had better diagnostic performance, with higher specificity, accuracy, and AUC. CAD can help recognize benign lesions, especially in patients with BI-RADS 4A, and avoid unnecessary invasive procedures.

## Highlights

• Computer-aided diagnostic (CAD) system correctly downgraded BI-RADS 4A patients.
• Revised BI-RADS category based on CAD could avoid unnecessary invasive procedures.
• CAD has better diagnostic performance than conventional ultrasound on breast masses.
• The effects are consistent in patients with smaller masses of ≤20 mm.

## 1 Introduction

According to the Global Cancer Observatory, breast cancer is the most prevalent cancer worldwide, with approximately 2.26 million new cases in 2020, and it is the fifth leading cause of cancer-related deaths [1]. It is estimated that approximately 429,105 newly diagnosed breast cancer cases and 124,002 breast cancer deaths were reported in China in 2022 [2]. Although cancer treatments are rapidly developing, the burden of breast cancer remains huge, both in China and worldwide. Highly effective diagnostic techniques for early breast cancer are essential to prevent disease progression and cancer-related deaths. For patients with breast lesions, correctly identifying benign and malignant breast diseases and providing appropriate treatment are crucial. Ultrasound (US) is one of the most employed cost-effective and non-invasive imaging modalities for breast examination. However, the role of US is controversial, and it is often used as an adjunctive tool in high-risk women [3]. This is due to the relatively high false-positive rate and, subsequently, the increased possibility of an unnecessary biopsy or surgery [4]. Another problem with US is that it is a highly operator-dependent technique [5]. Nevertheless, unlike Western countries, US plays an important role in detecting breast cancer among Chinese patients [6]. In addition to factors such as the lack of accessibility to mammography equipment and expertise, sociocultural barriers, and insufficient health education [7], Asian patients commonly presents with relatively smaller and denser breast tissue, which complicates mammography-based diagnosis [8,9]. Although the risk of overdiagnosis and overtreatment is a concern when implementing conventional US breast cancer screening and diagnosis, the newly developed artificial intelligence (AI) technology in the US may overcome some drawbacks. In recent years, deep-learning-based computer-aided diagnostic (CAD) systems (S-Detect) have been used to improve the diagnostic performance of breast US. Several studies have shown that CAD increases the accuracy of breast US in diagnosing breast cancer [[10], [11], [12], [13]]. However, this technology is relatively new, and more evidence is needed to establish its feasibility. This study aimed to examine the performance of CAD compared to conventional US and investigate its effects on smaller mass size groups.

## 2 Material and methods

### 2.1 Study design and patient selection

This was a retrospective analysis of patients with breast masses at Gangkou Hospital (Heibei, China) from March 2021 to July 2023. Patients who underwent breast ultrasound examinations and subsequent surgery or biopsy were included. The exclusion criteria included pregnancy, lactation, and other tumor diagnosis. All patient data were collected through a chart review. A total of 123 patients were included in this analysis. This study was reviewed and approved by the ethics committee of Gangkou Hospital, with the approval number: M2021304. Written informed consent was obtained from all patients.

### 2.2 Measurement and outcome

Histopathological results from ultrasound-guided core needle biopsies (18-Gauge) or surgical excisions are the gold standard for diagnosis. Patients’ clinical data were entered and collected by clinicians in the electronic health medical system. US examinations were performed by experienced radiologists using a US system (W10 machine) with S-Detect AI technology (version 1.02.00b.3006; Samsung Medison Co., Ltd., Seoul, South Korea). The breast masses were categorized according to BI-RADS by radiologists: Category 0: Incomplete; Category 1: Negative with 0 % likelihood of malignancy; Category 2: Benign with 0 % likelihood of malignancy; Category 3: Probably benign with ≤2 % likelihood of malignancy; Category 4A: Low suspicion for malignancy with >2 % to ≤10 % likelihood of malignancy; Category 4B: Moderate suspicion for malignancy with >10 % to ≤50 % likelihood of malignancy; Category 4C: High suspicion for malignancy >50 % to <95 % likelihood of malignancy; Category 5: Highly suggestive of malignancy with ≥95 % likelihood of malignancy. Breast masses were assessed using CAD and subsequently divided into two groups: possibly benign and possibly malignant. A revised BI-RADS category was provided by radiologists after considering the CAD results.

### 2.3 Statistical analysis

Clinical data analysis involved reporting means with standard deviations or medians with 25th and 75th percentiles for continuous variables and frequencies with proportions for categorical variables. Categorical and continuous variables were analyzed using the chi-square test and *t*-test, respectively. Sensitivity, specificity, positive likelihood ratio (PLR), negative likelihood ratio (NLR), and accuracy were calculated to evaluate diagnostic performance. The areas under the receiver operating characteristic curves (AUC) of conventional US and CAD were analyzed and compared using the Delong method. Cohen's kappa coefficient was calculated by the inter-rater agreement test: almost perfect (0.81–1.00), substantial (0.61–0.80), moderate (0.41–0.60), fair (0.21–0.40), none to slight (0.01–0.20), and no agreement (≤0). Logistic regression was used for the univariate regression. A two-sided P-value <0.05 was considered statistically significant. SAS 9.4 was used to perform all statistical analyses.

## 3 Results

### 3.1 Patients’ characteristics

Between March 2021 and July 2023, 123 patients with breast mass were treated at our hospital. The demographic and lesion characteristics are summarized in Table 1. The patients were predominantly female (95.12 %), with an average age of 52 years. Forty-three masses had smaller tumor sizes (7.32 % size≤10 mm, 27.64 % 10 mm < size≤20 mm). Most masses presented with one or more abnormalities on US: Not circumscribed margin (44.72 %), irregular shape (34.15 %), spiculation (25.2 %), calcification (14.63 %), moderate-high blood flow (5.69 %), and surrounding change (19.51 %).

**Table 1:** Patients' characteristics.

	All Patients (n = 123)
Age (years old)	52.02 ± 14.69
Age≥50	71(57.72 %)
Female	117(95.12 %)
BMI	24.26 ± 3.91
Overweight	31(25.2 %)
Obesity	7(5.69 %)
Tumor Size	
10 mm < size≤20 mm	34(27.64 %)
size≤10 mm	9(7.32 %)
Breast Density	
Low	105(85.37 %)
Medium	17(13.82 %)
High	1(0.81 %)
Margin-Not circumscribed	55(44.72 %)
Irregular Shape	42(34.15 %)
Spiculation	31(25.2 %)
Calcification	18(14.63 %)
Blood flow	
Minimal flow	17(13.82 %)
Moderate-High flow	7(5.69 %)
Surrounding Change	24(19.51 %)
BI-RADS category^a^	
3	59(47.97 %)
4A	43(34.96 %)
4B	14(11.38 %)
4C	6(4.88 %)
5	1(0.81 %)
Revised BI-RADS category^b^	
3	89(72.36 %)
4A	9(7.32 %)
4B	11(8.94 %)
4C	13(10.57 %)
5	1(0.81 %)

Values are means ± SD or n (%).

a Based on conventional ultrasound.

b Based on conventional ultrasound and computer-aided diagnostic system.

The pathological diagnosis and findings are summarized in Table 2. Ninety-six patients were diagnosed with benign disease, while 27 patients were diagnosed with malignant breast cancer (including 4 ductal carcinomas in situ, 22 invasive ductal carcinomas, and 1 mucinous breast cancer).

**Table 2:** Pathology diagnosis and findings.

Diagnosis (n = 123)	n = 123
**Malignant**	27(21.95 %)
Ductal carcinoma in situ	4(3.25 %)
Invasive Ductal Carcinoma	22(17.89 %)
Mucinous breast cancer	1(0.81 %)
**Benign**	96(78.05 %)
Fibroadenoma	56(45.53 %)
Intraductal papilloma	30(24.39 %)
Fibrocystic breast disease	5(4.07 %)
Duct ectasia	18(14.63 %)
Apocrine Metaplasia	8(6.5 %)
Epithelial Hyperplasia	11(8.94 %)
Adenosis	6(4.88 %)
Acute suppurative mastitis	1(0.81 %)
Gynecomastia	3(2.44 %)
**Biomarkers (n = 52)**	
ER (+)	45(86.54 %)
PR (+)	41(78.85 %)
HER-2 (+)	10(19.23 %)
Ki67 (+)	44(84.62 %)
Ki67%^a^	2(1–12.5)
<15 %	33(75 %)
≥15 %	11(25 %)

Values are n (%) or Median (Lower Quartile-Upper Quartile).

a n = 44.

### 3.2 Diagnostic performance comparison between conventional US and CAD (all patients and sizes ≤20 mm)

**Fig. 1:**
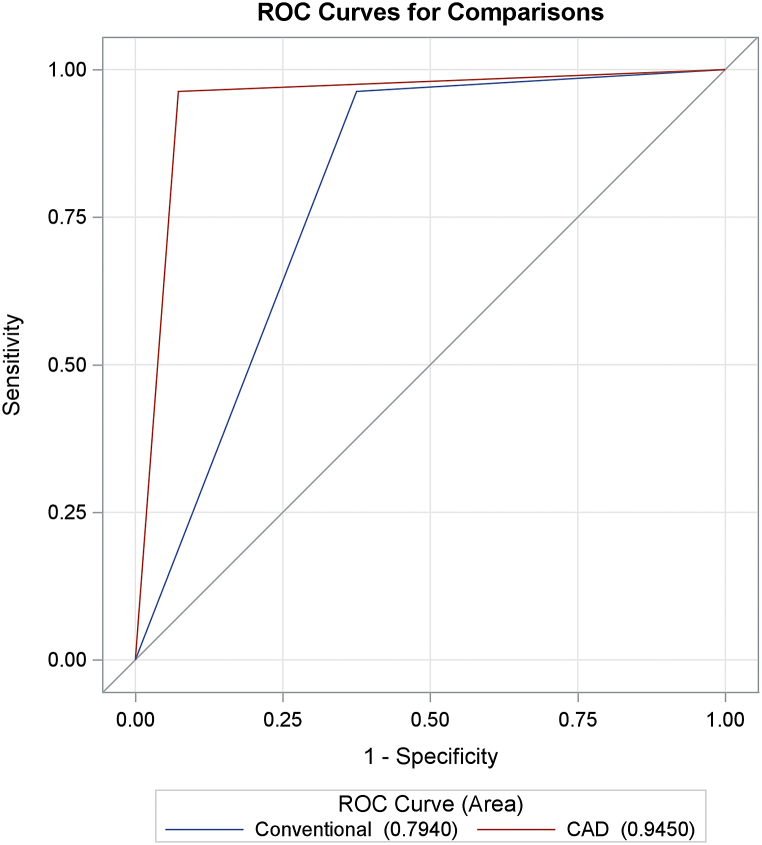
ROC Curves of Conventional US and CAD (All Patients) Abbreviation: ROC: receiver operating characteristic curve; US: ultrasound; CAD: computer-aided diagnostic system.

**Fig. 2:**
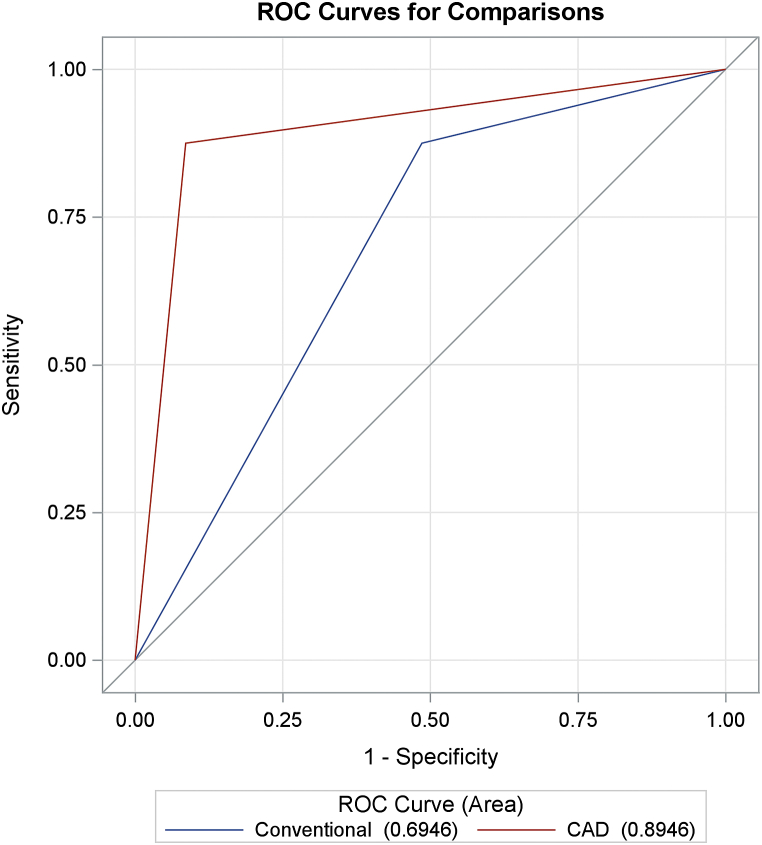
ROC Curves of Conventional US and CAD (size≤20 mm) Abbreviation: ROC: receiver operating characteristic curve; US: ultrasound; CAD: computer-aided diagnostic system.

Table 3 and Fig. 1 show the diagnostic performances of conventional US and CAD. Both conventional US and CAD had high sensitivity (>95 %), but CAD had much higher specificity (92.71 % vs. 62.50 %) and accuracy (93.50 % vs. 69.92 %). The AUC for CAD was also significantly higher than that for conventional US (0.9450 vs. 0.7940, p < 0.0001). The agreement (Table 4) between the CAD and pathology results was almost perfect (kappa = 0.82, p < 0.0001), whereas conventional US only had fair agreement with the pathology results (kappa = 0.4, p < 0.0001). The effect was consistent in patients with a mass size of ≤20 mm (Table 5 and Fig. 2): CAD had higher specificity (91.43 % vs. 51.43 %), higher accuracy (90.70 % vs. 58.14 %), and a greater AUC (0.8946 vs. 0.6946, p < 0.0001) than conventional US.

**Table 3:** Diagnostic performances of conventional US and CAD.

	Sensitivity (%)	Specificity (%)	PPV (%)	NPV (%)	Accuracy	AUC*
Conventional US	96.30 %	62.50 %	41.94 %	98.36 %	69.92 %	0.7940
CAD	96.30 %	92.71 %	78.79 %	98.89 %	93.50 %	0.9450

*P < 0.0001. Abbreviation: US: ultrasound; CAD: computer-aided diagnostic system.

PPV: positive predictive value; NPV: negative predictive value; AUC: area under curve.

**Table 4:** Agreement between US and pathology.

	kappa	P-value	Agreement
Conventional US vs. Pathology	0.4	<0.0001	fair
CAD vs. Pathology	0.82	<0.0001	almost perfect

Abbreviation: US: ultrasound; CAD: computer-aided diagnostic system.

**Table 5:** The diagnostic performances of conventional US and CAD (size≤20 mm).

	Sensitivity (%)	Specificity (%)	PPV (%)	NPV (%)	Accuracy	AUC*
Conventional US	87.50 %	51.43 %	29.17 %	94.74 %	58.14 %	0.6946
CAD	87.50 %	91.43 %	70.00 %	96.97 %	90.70 %	0.8946

*P < 0.0001. Abbreviation: US: ultrasound; CAD: computer-aided diagnostic system.

PPV: positive predictive value; NPV: negative predictive value; AUC: area under curve.

### 3.3 Changes in the BI-RADS category based on CAD

**Fig. 3:**
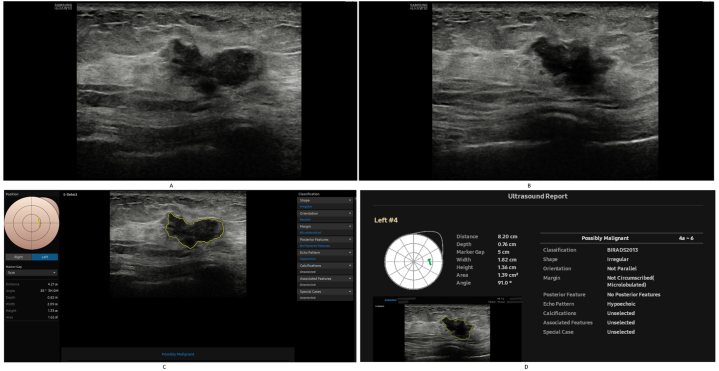
74-year-old woman diagnosed with invasive ductal carcinoma. A-B. Conventional ultrasound showed: on 2D ultrasound, a hypoechoic area is visible in the 2–3 o'clock direction of the upper outer quadrant of the left breast (images from 85° to 91° angle), with an approximate size of 1.4–1.6 cm^2^. The morphology is irregular, and the margin is indistinct. Radiologist classified as BI-RADS 4B. C.CAD description screen (based on 85° angle image): The shape is irregular. The margin is microlobulated. CAD Diagnosis: possibly malignant. D. CAD Report screen (based on 91° angle): The shape is irregular. The orientation is not parallel. The margin is not circumscribed (microlobulated). CAD Diagnosis: possibly malignant. Radiologist reassessed it as category 4C based on multiple CAD reports.

Table 6 presents the revisions of the patients’ BI-RADS categories after receiving the CAD results. A total of 42 changes occurred, all of which were originally 4A or 4B. Among the 35 changes in 4A patients, all 29 downgrades were proven benign, and all six upgrades were proven malignant. Similarly, among the seven changes in 4B patients, both two downgrades were proven benign, and all five upgrades were proven malignant. Fig. 3 presents the ultrasound images of an invasive ductal carcinoma case, which was originally BI-RADS 4B and was upgraded to 4C based on the CAD results. The radiologist commented that “the morphology is irregular, and the margin is indistinct” on the conventional ultrasound images, and CAD added information of “Orientation: Not Parallel; Margin: Not Circumscribed (Microlobulated)” and “Possibly Malignant.”

**Table 6:** Changes in BI-RADS category based on CAD.

Changes	n = 42	Pathology
**Downgrades**		
4A-3	29	Benign
4B-3	1	Benign
4B-4A	1	Benign
**Upgrades**		
4A-4B	4	Malignant
4A-4C	2	Malignant
4B–4C	5	Malignant

Abbreviation: CAD: computer-aided diagnostic system.

### 3.4 Association between biomarkers and CAD-predicted malignancy

Table 7 shows the associations between biomarkers and CAD results. HER-2 (+) was significantly associated with CAD-predicted malignancy (OR = 5.65, p = 0.0421), but no relationships were found between CAD results and ER/PR status or Ki-67 status.

**Table 7:** Association between biomarkers and CAD predicted malignancy.

	ER/PR (+)	HER-2 (+)	Ki-67
CAD (+)	0.12(0.01–1.10)	5.65(1.06–29.98)	3.29(0.60–18.10)
P value	0.0605	0.0421	0.1718

Values are OR (95%CI). Associations were analyzed using logistic regression.Abbreviation: CAD: computer-aided diagnostic system.

## 4 Discussion

US is one of the most used tools for breast tumor detection in China. However, it has also been criticized for its relatively elevated false-positive rate and dependency on experienced radiologists. CAD systems can overcome these drawbacks and improve the diagnostic capability of breast US. In the current study, we analyzed 123 patients with breast masses who were suspected of having breast cancer. The pathology results were used as the gold standard to examine the efficacy of CAD-enhanced breast US in detecting breast cancer.

Our results showed that conventional US had high sensitivity but relatively low specificity and accuracy, whereas CAD exhibited an overall high sensitivity, specificity, accuracy, and AUC (96.3 %, 92.71 %, 93.5 %, and 0.945, respectively). These results were consistent with those of previous studies, with CAD sensitivity ranging from 85.5 to 95.8 %, specificity ranging from 72.27 to 93.8 %, accuracy ranging from 80.45 to 89.6 %, and AUC ranging from 0.807 to 0.948 [[13], [14], [15], [16], [17], [18]]. The agreement between the CAD and pathology results was almost perfect, with only 8 (6.5 %) cases being inaccurately predicted by CAD (including 1 false-negative and 7 false-positive cases). Misdiagnosis may be related to factors such as the inability to identify microcalcifications or suboptimal image quality of distant lesions. Furthermore, we conducted a subgroups analysis on smaller masses, which underscored CAD's sustained efficacy in diagnosing masses ≤20 mm. Xing et al. reported that the addition of CAD technology to conventional US imaging could better differentiate benign from malignant small breast nodules ≤20 mm [13]. Liang et al. also confirmed that the AUC of combined-CAD mode in lesions ≤20 mm is better than without-CAD mode in both novice and experienced readers [19]. Collectively, CAD exhibited markedly superior diagnostic performance compared to conventional ultrasound.

In our study, radiologists predicted malignancy in 62 cases using conventional US; however, less than half of the cases were pathologically proven to be malignant. This yielded a total of 36 false-positive results. Most false-positive cases were 4A cases (34 4A cases and 2 4B cases), as clinicians might tend to be overly cautious about the possibility of missing potential cancers when dealing with patients in the ‘middle ground.’ After obtaining possible benign/malignant results from CAD, a revised BI-RADS category was provided by the radiologists. All revisions were made to the original 4A and 4B patients, and these changes were pathologically proven to be appropriate. CAD showed high overall accuracy and successfully helped the radiologists classify 4A and 4B patients into the correct groups. Zhu et al. found that category 4A and 4B lesions were more likely to have discordant CAD results than lesions in other categories [20]. Two studies also showed that AI technology could help reduce unnecessary biopsies by downgrading 4A lesions [10,21]. Other researchers have reported that CAD can significantly improve diagnostic accuracy, especially for novice readers [[15], [16], [17], [18]]. Although 59 original BI-RADS 3 patients and 89 revised BI-RADS 3 patients were included in our study, they still underwent biopsies or surgery. This might be due to 1) consideration of other clinical factors (history, age, symptoms, and other abnormal test results), 2) some clinicians being overly cautious or less confident, and 3) still being in the transition of adapting new AI technology. If CAD results are considered a factor during the decision-making process, at least a quarter of our patients could avoid invasive procedures. Based on the current findings, we recommend that health practitioners be more confident about making decisions among patients in the lower categories using CAD results to prevent unnecessary invasive procedures. CAD can also be used to train and provide support to inexperienced young readers.

Another subgroup analysis of patients with biomarker results (ER/PR, HER-2, Ki67) was performed. The results showed that HER-2 positivity was significantly associated with CAD-predicted malignancies, whereas ER/PR (+) and Ki-67(+) were not. When we investigated the details of these false-positive patients with CAD among those with biomarker results, ER/PR (+) and/or Ki-67(+) were present in all cases. A previous study found that Ki-67 status was significantly associated with discordant CAD results [20]. Whether patients with ER/PR (+) and/or Ki-67(+) had any particular change on ultrasound in the very early phase remains a question, and we lacked sufficient data to analyze it and draw any conclusions. Further investigations are required to address this question.

This study had several limitations. First, it was conducted at a single center with a relatively small sample size. Given the novelty of the technology, only patients within the last two years were included, which consequently limited our ability to perform additional analysis on further questions. Second, since the patients were at various disease stages and required different interventions, the pathology results used as the gold standard were not consistently derived solely from surgery or core needle biopsy throughout the study population. Third, CAD currently cannot achieve comprehensive, intelligent diagnosis. Calcifications, blood flow, surrounding changes, and lymphatic enlargement were set as “unknown” by default. Furthermore, it may not be able to differentiate pathological changes from hormone-influenced physiological changes due to pregnancy and lactation. In the future, multicenter, large-scale studies with more comprehensive data are required to address these questions.

## 5 Conclusions

Compared to conventional breast US, CAD has better diagnostic performance, with higher specificity, accuracy, and AUC. The agreement between CAD results and pathologic results was favorable. CAD can help recognize benign lesions, especially in patients with BI-RADS 4A, and avoid unnecessary invasive procedures.

## Ethics statement

This study was reviewed and approved by the ethics committee of Gangkou Hospital, with the approval number: M2021304. All patients provided informed consent to participate in the study. All patients provided informed consent for the publication of their anonymised case details and images.

## Data availability statement

Data will be made available on request.

## Additional information

No additional information is available for this paper.

## CRediT authorship contribution statement

**Pengjie Song:** Writing – original draft, Software, Resources, Methodology, Formal analysis, Conceptualization. **Li Zhang:** Software, Investigation, Formal analysis. **Longmei Bai:** Project administration, Data curation. **Qing Wang:** Visualization, Software. **Yanlei Wang:** Writing – review & editing, Supervision.

## Declaration of competing interest

The authors declare that they have no known competing financial interests or personal relationships that could have appeared to influence the work reported in this paper.
